# The role of structured exercise interventions on cognitive function in older individuals with stable Chronic Obstructive Pulmonary Disease: A scoping review

**DOI:** 10.3389/fresc.2022.987356

**Published:** 2022-10-31

**Authors:** Caroline C. Eastus, Daniel E. Baez, Maria L. Buckley, Jungeun Lee, Alessandra Adami

**Affiliations:** ^1^ Department of Kinesiology, College of Health Sciences, University of Rhode Island, Kingston, RI, United States; ^2^ Department of Psychiatry and Human Behavior, Warren Alpert Medical School of Brown University, Providence, RI, United States; ^3^ College of Nursing, University of Rhode Island, Kingston, RI, United States

**Keywords:** smokers, cognitive impairment, pulmonary rehabilitation, aging, mental health, physical activity

## Abstract

A decline in cognitive performance has been associated with disease severity, exacerbations rate, presence of comorbidities, and low activity level in people with chronic obstructive pulmonary disease (COPD). Participation in exercise programs appears to have neuroprotective effects and to improve cognitive performance in older people. The present work undertook a scoping review of the effects of exercise-based interventions on cognitive function in older individuals with stable COPD.

**Methods:**

The methodological framework for scoping review was used and electronic searches of five databases performed. Original research and observational studies published between January 2010 and December 2021, administering exercise-based interventions and cognitive function evaluation, were included.

**Results:**

Of 13 full-text manuscripts assessed for eligibility, five were allocated to analysis. Three studies administered exercise training within pulmonary outpatient rehabilitation program (PR), and one inpatient PR. The fifth study conducted a structured training intervention in which either aerobic or a combination with resistance exercises were included. Twelve cognitive function screening tools were used in the five studies included in the analysis. Results extracted were based on 245 COPD (33% female) with moderate to very-severe airflow limitation. Interventions ranged from 12 to 36 sessions. Studies reported statistically significant improvements after intervention in different cognitive function domains, such as global cognition, immediate and delayed recall ability, cognitive flexibility, verbal fluency, attention, abstract reasoning, praxis ability.

**Conclusions:**

Exercise-based interventions improve several areas of cognitive function in patients with stable COPD. However, the magnitude of gain varies among studies, and this is possibly due to the heterogeneity of tests used. Future research is needed to validate the optimal battery of screening tests, and to support the definition of guidelines for cognitive function evaluation in COPD.

## Introduction

Chronic obstructive pulmonary disease (COPD) is a common, preventable, and treatable disease characterized by persistent respiratory symptoms and airflow limitation (1), which is usually progressive (2) and associated with a poor quality of life and increased hospitalization, morbidity, and mortality rates (3). While COPD is primarily a respiratory disease, great attention has been paid to identify and clarify the role of extrapulmonary complications (e.g., skeletal muscle dysfunction, malnutrition, osteoporosis) in progressively impacting the symptoms and quality of life in patients (4). Recent studies indicated that cognitive impairment is an extrapulmonary complication with a prevalence of 10%–61% among patients with COPD (5–8), which is a higher rate than the general older adult population (3%–20%), confirming that a correlation exists between impaired pulmonary and cognitive function (9, 10). Studies reported that in representative sample of the US older population, 17%–25% of the individuals with dementia have a concurrent diagnosis of COPD (11, 12); and that the co-existence of these two conditions has an additive effect on respiratory-related and all-cause of hospitalizations, and morbidity, in the COPD population (13).

Cognitive impairment (CI) or dysfunction, which reflects a performance that is lower than expected for an individual in relation to group norms, is defined as the difficulty remembering, learning new information, concentrating, and with decision making (9). Confusion and impaired judgement appear generally at an early stage, either singularly or in combination, and often proceed an inability to perform movements and coordination that are revealed when the CI has significantly progressed (9). In COPD, the risk of developing cognitive dysfunction is positively associated with the progression of the severity of the disease (14), the frequency of exacerbations, the presence of comorbidities, and negatively with the level of education (15). Moreover, compared to their peers, patients with COPD appear to be at an increased risk for mild CI (MCI) (16), a state that falls between normal age-related decline and dementia (17), which is not always progressive (18). Neuropsychological deficits in patients with COPD appear to impact attention, memory, cognitive flexibility (19, 20), and speech (20). A loss in these cognitive skills may limit the ability to initiate, organize and execute essential self-care functions and to properly follow the daily treatment therapy (13). In COPD, the risk factors attributed to CI are the presence of inflammation and oxidative stress; and of comorbidities such as diabetes, hypertension, heart disease and cancer (10, 21). Likewise, sedentary lifestyle and tobacco smoking have been associated with impaired cognitive ability in COPD (9). While specific pathophysiological mechanisms are not clearly understood, it is suggested that, especially in middle-age and older smokers, the concomitant presence of inflammation, oxidative stress and lack of physical activity can accelerate the aging process and worsen age-related cognitive deficits (22). Multiple pathophysiological factors have been associated with COPD and neuropsychological impairment, such as the decrease in oxygen supply due to chronic airflow limitation (9), the exposure to frequent hypoxemia (19) causing brain ischemic damage, grey and white matter deterioration (14, 23, 24), loss of cerebral neurons (25), and presence of brain amyloid beta plaques, a hallmark feature of MCI (26). The COPD population appears to have also a higher prevalence of cerebral microbleeds (27), consequence of arteriosclerotic processes (28) and marker of small vessel diseases (27), and a reduction in hippocampal volume (29) caused by chronic hypoxemia (29, 30).

Regular physical activity and exercise training promote the maintenance and improvement of cognitive performance (5, 31, 32) and has neuroprotective effects (33). In COPD, exercise training is commonly but not exclusively delivered as part of pulmonary rehabilitation (PR), a multidisciplinary program aiming to improve physical and psychological conditions and educate about the benefits of regular physical activity (34, 35). However, the gain derived from regular exercise training on cognitive function in the COPD population is poorly defined. To date, two studies sought to review the effects of exercise on cognitive performance in patients with COPD (33, 36) suggesting that exercise might be beneficial, but evidence is still limited. Neither review provided a description of the cognitive function tests used or quantified the magnitude of post intervention changes in patients with COPD. Therefore, the aim of this scoping review was twofold: (1) to provide a more thorough description of the types of cognitive function tests used to screen patients in concomitance of the participation in exercise-based interventions; and (2) to calculate and compare the magnitude of changes induced by these interventions on cognitive abilities, in older adults with stable COPD. Accordingly, this review question was twofold: what is known from the literature about the role structured exercise-based programs have on the cognitive function in older people with a diagnosis of stable COPD? And which instruments have been selected to evaluate cognitive function in the COPD population?

## Methods

The methodological framework proposed by Tricco et al. (37) and Peters et al. (38) for conducting scoping review was followed for preparing this work. Manuscripts search was performed using the following databases: PubMed/MEDLINE, EMBASE, Science Direct, Cochrane Library, and Cumulative Index to Nursing and Allied Health (CINAHL). Pulmonary rehabilitation practice guidelines (39–41) and pulmonary professional guidelines (42) have evolved over the past decade, thus our literature search was limited to original research and observational studies published between January 2010 and December 2021 (i.e., date of the most recent search was executed). Additional manuscripts were sought through cross-referencing. The key terms and concepts used in the search, and the strategy, are available as Supplementary Materials (Supplementary Table S1 and Supplementary S2, respectively).

### Study selection

Two authors (CCE, DEB) independently searched the databases. Search concepts included: COPD, Smokers, Physical Activity, and Cognitive function (complete list of search terms available in Supplementary Table S1). Studies were included if they: (1) enrolled a cohort of older adults (≥65 years old) with stable COPD; (2) included an exercise-based intervention; (3) evaluated cognitive function through questionnaires or medical assessments (e.g., brain imaging scans). Studies were excluded if: (1) published before 2010; (2) written in a language other than English; (3) daily physical activity or exercise were measured using questionnaires; (4) were case-technical study reports, reviews, or gray literature (no commercial or academic publishing material e.g., government reports, white papers).

The search results were imported into Microsoft Excel and the same authors (CCE, DEB) reviewed the titles and abstracts to remove articles that did not meet inclusion criteria. If the content from the abstract was unclear, articles were included for a subsequent full manuscript review. After preliminary screening, the same two authors independently inspected the full texts, according to the inclusion/exclusion criteria. Any articles in question were discussed with the fifth author (AA) and resolved by consensus. Furthermore, reference lists of the included articles were reviewed to identify additional eligible papers that might have been missed during the first round of search.

### Data extraction

Extracted information included study design, population characteristics (cohort size, age, sex, pulmonary function, presence of CI), main characteristics of the exercise interventions (type of training, length, exercise routine, workload and progression, session duration), and instruments used to evaluate cognitive function.

### Data synthesis

A descriptive synthesis was performed for the outcomes of interest and reported in tabularized format (Table 1). The changes in cognitive performance after the intervention were calculated as the difference between the absolute values at pre and post study phase by two independent authors (CCE, AA) (Table 3). Only one study (43) did not provide post exercise-intervention absolute values (e.g., mean ± SD, median, range) for the cohort. Therefore, changes were calculated by adding to the group mean at pre intervention the value of the coefficient authors provided for their longitudinal analysis (Table 2 pertaining to Pereira et al. (43)), while the standard deviation was calculated by multiplying the standard error by the square root of the cohort sample size.

**Table 1:** Description of the studies included in this review.

Study (Ref. #)	Design	Cohort characteristics	Intervention	Cognitive Function Instruments
Pereira et al. 2011 (43)	Prospective observational study	Two groups: • 34 COPD (GOLD 1/2/3/4 = 0/11/15/8) patients: - Age: 65.2 ± 7.0 years - 50% female • 18 healthy age and sex-matched individuals from community senior center: - Age: 62.7 ± 4.0 years - 50% female	• Multidisciplinary outpatient PR. Program components: exercise training, educational, psychosocial sessions • 3 times/week, 3 months, 36 sessions total • Neuropsychological evaluation administered at baseline and 3 months following PR	• The Stroop test • F-A-S Test • The Digit Span test • RAVLT test
Aquino et al. 2015 (44)	Randomized control trial	• 28 former smokers, Caucasian male COPD: - Age: 67.2 ± 7.9 years - FEV_1_/FVC: 62.1 ± 8.2 - FEV_1_%pred: 68.4 ± 11.5 • Randomized in 2 groups: (1) Combined group (aerobic and resistance training); *n* = 14 (2) Aerobic Training (AT); *n* = 14	• Two 30-min training sessions per day (AM and PM), 5 days/week, 4 weeks • Resistance training (30 min): 3 sets, 12 repetitions, from 70% 1-RM (week 1) to 90% 1-RM (week 4) for deltoids, biceps, dorsal muscles, quadriceps. Work rate increase based on a progressive reduction from 10 to 4 repetitions/set, while maintaining 3 sets/exercise • Endurance training (40 min): 5 min warm up (walking on treadmill at 35% V˙O_2max_ based on CPET done at intake), 30 min training (on treadmill), 5 min cooldown (stretching). Work rate intensity for training phase progressed from 70% HR_max_ (week 1) up to 90% HR_max_ (week 4). Tolerance to effort was constantly monitored using HR monitors and RPE (Borg scale).	• Rey 15-item memory test • Drawing copy test • Attentive matrices test • Raven's progressive matrices test • Verbal fluency test
Bonnevie et al. 2020 (45)	Prospective observational study	• 56 COPD patients referred to PR: - Age: 62.0 ± 9.0 years - 54% female - FEV_1_ (L): 0.9 (0.7−1.1) - FEV_1_/FVC: 41.0 ± 10.0 - FEV_1_%pred: 36.0 (28–44) - 73% with diagnosis of CI	• Multidisciplinary outpatient PR: 3 times/week, 8 weeks, 24 sessions total. Program components: respiratory physiotherapy, muscle strengthening, endurance training, self-management, nutrition • Resistance training: 3 sets, 12 repetitions, at 70% 1-RM. Use of free weights and elastic bands • Endurance training: 5 min warm-up, progressive exercise (from 15-to-45 min), 5 min cooldown. Work rate initially calculated on anaerobic threshold determined during CPET done at intake. Work rate and exercise duration progression was based on individual RPE (Borg scale).	• MoCA (three versions of the test were used to prevent learning effects, in a cross-over randomized order)
France et al. 2021 (46)	Prospective, observational study	• 67 stable COPD: - Age: 68.5 ± 6.4 - 45% female - 26% current smokers - FEV_1_/FVC: 55.0 ± 18.0 - FEV_1_%pred: 54.0 (38.5-72.5) - 57% with diagnosis of CI (*n* = 36 mild CI; *n* = 2 moderate CI)	• Multidisciplinary outpatient PR: 2 times/week, 6 weeks, 12 supervised sessions: - 1 h aerobic and resistance exercise training (based on British Thoracic Society guidelines)	• MoCA
Andrianopoulos et al. 2021 (13)	Prospective, observational study	• 60 stable COPD: - Age: 67.7 ± 8.4 - 25% female - 8% current smokers - FVC %pred: 69.7 ± 18.0 - FEV_1_%pred: 46.7 ± 15.4 - 42% with diagnosis of CI	• Multidisciplinary inpatient, supervised PR: 4 times/week, 3 weeks, 12 sessions total. Program components: exercise training (80 min), education on COPD self-management, physical activity counseling • Exercise training (50 min): - Endurance training: 20 min bike/treadmill, 60%–70% peak work rate. Work rate initially calculated during CPET done at 75% of individual estimated peak work rate, on a cycle ergometer at intake. Work rate and exercise duration progression was based on individual RPE for dyspnea and leg fatigue symptoms (Borg scale). - Resistance training: 3 sets, 15 repetitions, 6 exercises (leg press, knee extension, hip abduction/adduction, shoulder pull down, rowing, abdominal), individual load (aim to reach momentary muscle fatigue by end of set evaluated by 0-10 Borg RPE scale) • Physical activity (30 min): - low-to-moderate individual exertion - walking and/or calisthenics exercises using body weight, small dumb-bells, rubber tubes	• SMMSE • ACE-R (v.2007) • MoCA • T-ICS

Data are reported as mean ± SD, or median(IQR range). COPD, Chronic Obstructive Pulmonary Disease; GOLD, Global Initiative for Chronic Obstructive Lung Disease; FEV_1_, forced expiratory volume within 1 s; FVC, forced vital capacity; CI, cognitive impairment; PR, Pulmonary Rehabilitation; F-A-S test, Verbal Fluency Test; RAVLT, The Rey Auditory Verbal Learning Test; CT, Combined training; AT, Aerobic training; 1-RM, 1-Repetition Maximum; V˙O_2max_, maximal oxygen consumption; HR_max_, maximal heart rate; HR, heart rate; RPE, Borg's Ratings of Perceived Exertion; CPET, cardiopulmonary exercise test; MoCA, Montreal Cognitive Assessment; SMMSE, Standardized Mini-Mental Status Examination; ACE-R, Addenbrooke's Cognitive Examination-Revised; T-ICS, Interview for Cognitive Status (administered face-to-face).

**Table 2:** List of the neurocognitive tests administered in the five studies allocated to analysis.

Test	Cognitive function domains and processes	Description and Reference source
Montreal Cognitive Assessment, MoCA	Visuospatial ability, executive functions, language, short-term memory, attention and concentration, working memory, orientation and place	Test requires completion of 30 tasks in approximately 10-15 min. It is a paper-and-pencil screener for mild cognitive impairment assessing multiple domains. Each task, that correspond to a cognitive ability, is assigned a value: 6-point for orientation and place; 5-point for two learning trials of five nouns and delayed recall; 3-point for clock drawing task and three item confrontation naming task with familiar animals; 2-point for 2-item verbal abstraction and repetition of two syntactically complex sentences; 1-point for three-dimensional cube copy, trail-making task, phonemic fluency task, attention task (tapping), digits forward and backward. Maximal score is of 30 points. Diagnosis is based on the score obtained, where: – >26 indicating normal cognition – 18–25 mild cognitive impairment – 10–17 moderate cognitive impairment – <10 severe cognitive impairment Test has an 81% sensitivity, 72% specificity, 76% correct diagnosis rate, when administered for detection of cognitive impairment in COPD patients. Nasreddine et al., 2005 (47); Crisan et al., 2014 (48); Villeneuve et al., 2012 (49)
Standardized Mini-Mental Status Examination, S-MMSE	Orientation, short-term memory, recall, registration, constructional ability, language, comprehension and command execution ability	Test is a 10-minute screener for cognitive impairment. The examiner and the responder sit facing each other, and the examinee completes a series of different tasks to target the cognitive function domains under investigation, e,g. spelling backward, drawing, folding piece of paper, writing. Score assigned ranges from zero to a maximum of 5 (e.g., up to 0–5 for spelling task, 0-1 for drawing). The maximum score is of 30 points, that is subsequently adjusted and rounded before final score is confirmed. The score defines the stages of the disease: – 25–30 indicating normal cognition – 21–24 mild/early cognitive impairment – 10–21 moderate cognitive impairment – 0–9 severe cognitive impairment Molloy et al., 1991 (50)
Addenbrooke's Cognitive Examination-Revised, ACE-R	Attention/orientation, memory, verbal fluency, language, visuospatial ability	Test is a brief 15–20 min test battery and it contains five subsets, each one representing a cognitive function domain. The testee completes a series of 11 tasks that refer to the specific subset, e.g. recall 3 words mentioned by examiner to assess memory recall ability, name the day/date/month/year/season to determine orientation. Each subset is scored separately: attention/orientation (18 points), memory (26 points), fluency (14 points), language (26 points), visuospatial (16 points). The maximum score is 100, derived by the sum of the five subsets. Mioshi et al., 2006 (51)
Interview for Cognitive Status, T-ICS	Orientation, memory, attention/concentration, language	The T-ICS is brief, 10-minute, standardized test designed to be administered over the phone or face-to-face. The examiner directs the responder to complete 11 cognitive domain specific tasks that, e.g. naming the date and responder's full name to evaluate orientation, recalling words read by examiner to assess verbal memory. The total score is 41 points, which reflects global cognitive functioning (a greater score indicates higher performance). Brandt et al., 1988 (52); 1993 (53)
The Rey Auditory Verbal Learning Test, Rey-test or RAVLT	Verbal learning and memory, inhibition, retention, subjective organization	Test is designed as a verbal list-learning paradigm. The participant is instructed to name a list of 15 words at two specific time points: (1) immediately after examiner listed the 15 words, five trials; (2) following 15 min delay. Each correct word recalled is valued at 1 point. Maximal score is equivalent to 75 points for the immediate recall; while a maximum score of 15 points for the delayed recall. Rey et al., 1959 (54)
The Oral Fluency Test, F-A-S Test	Phonemic fluency	This test required the responder to orally produce words that begins with the letters F, A, and S. Per each letter the responder has a minute to list as many words as possible. This tool is a subset of the Neurosensory Center Comprehensive Examination for Aphasia. Nutter-Upham et al., 2008 (55)
Verbal Fluency Test	Semantic Fluency	Test requires the responder to list in 2 min as many known words in four specific categories: colors, animals, fruits, name of cities. A 1-point value is given per each correct word. The total score is subdivided by four, and the scores range from 0 (very poor) to infinite (greater score indicates higher performance) Kaszniak et al., 1979 (56)
Digit Span	Attention, concentration, mental control, memory	The examinee repeats a sequence of numbers read by the examiner following the same (forward span) or the reverse (backward span) order. Score is calculated by the total number of correctly recalled digit spans from each trial, returning single scores based on the span typology and a summary evaluation. This instrument is a subset of Wechsler Adult Intelligence Scale, VIII. Blackburn et al., 1957 (57)
Attentive Matrices Test	Selective and sustained attention	Test consists of three numeric matrices, organized as ten columns of 13 numbers from 0 to 9. Responder is requested to check specific target numbers, from one to four digits, in 45 s for each matrix. The targets are embedded in each matrix: one target in the first matrix, two in the second, and three in the third. Per each correct target retrieve a 1-point value is assigned. The time necessary to complete the task is the basis for scoring. Spinnler et al., 1987 (58)
The Stroop Color and Word Test, Stroop test or SCWT	Selective attention, cognitive flexibility, processing speed	This test requires the responder to read three different tables as rapidly as possible. Two of these tables represent the “congruous condition” in which the participant is required to read names of colors printed in black ink (W task), and name different color patches (C task). Whereas in the third table color-words (CW task) are printed in an inconsistent color ink to test for an “incongruent condition”, for this task the participant must name the color of the ink instead of reading the word. Test subsets are scored in seconds above the predicted test duration, which is based on sex and education level (the length of time to complete the test associates with poorer performance). The overall aim of this test is to evaluate the ability to inhibit cognitive interference. Stroop, 1935 (59); Golden, 1975 (60); Scarpina & Tagini, 2017 (61)
Raven's Progressive Matrices Test	Abstract reasoning, problem solving	This is a multiple-choice test composed of a series of visual pattern matching and analogy problems in no representational designs. Test uses three series of 12 figures, for a total of 36 figures. Responder is instructed to retrieve the missing piece that composes a figure. Each correct answer has a value of 1 point, to reach a maximum score of 36 (score ranges from 0 – lowest - to 36 - highest). Raven, 2000 (62)
Drawing Copy Test	Praxis abilities	Tests requires completion of two drawing tasks. The simple copy task consists of copying three geometric drawings - a star, a cube, a house – as exactly as possible on the lower part of the sheet showing the figures. The examiner scores each drawing from 0 to 4; a greater score indicates higher performance. The second task consists of completing 10 geometrical drawings to obtain one of the figures used in the first task, with cueing. The 10 elements include: 2 copies of starts, 4 copies of cube and 4 of house. One point is assigned for each element (i.e., use of a line to complete in the missing elements in the given design). Maximum score is 70 (i.e., 70 missing lines in the 10 designs given). Gainotti et al., 1977 (13); Caltagirone et al., 1979 (63)

**Table 3:** Cognitive function tests results at the beginning (PRE) and end (POST) of the intervention, in the five studies allocated to analysis. Data referred to the COPD patients enrolled in each study, whose n is reported on the top of the table for the two phases of the intervention.

Cognitive Function Test	Phase	Pereira et al., 2011	Aquino et al., 2015			Bonnevie et al., 2020	France et al., 2021	Andrianopoulos et al. 2021	
			Whole cohort	CT	AT			NCI	CI
		*N*: pre = 34	*N*: pre = 28	*N*: pre = 14	*N*: pre = 14	*N*: pre = 56	*N*: pre = 67	*N*: pre = 35	*N*: pre = 25
		post = 34	post = 28	post = 14	post = 14	post = 37	post = 42	post = 35	post = 25
MoCA	PRE					22 (20, 26)	24.6 ± 3.2	27.4 ± 1.4	23.1 ± 2.5
	POST					25 (23, 28)**	26.2 ± 2.4*	27.9 ± 5.4^a^	23.3 ± 3.0^a^
S-MMSE	PRE							28.5 ± 1.1	27.0 ± 1.3
	POST							29.0 ± 0.8*	27.8 ± 1.2*
ACE-R	PRE							92.5 ± 3.6	81.7 ± 5.9
	POST							94.1 ± 3.0*	86.4 ± 5.1*
T-ICS	PRE							35.5 ± 1.9	32.6 ± 2.3
	POST							36.5 ± 1.6*	33.9 ± 2.3*
Rey test / RAVLT	PRE	*Σ*_1−5_: 43.5 ± 10.0 IR: 7.5 ± 3.3 DR: 8.4 ± 3.8	*Σ*_1−5_: 39.0 ± 9.5 IR: – DR: 7.6 ± 2.4	*Σ*_1−5_: 41.7 ± 10.5 IR: – DR: 8.3 ± 2.7	*Σ*_1−5_: 36.1 ± 7.8 IR: – DR: 7.0 ± 1.8				
	POST	*Σ*_1−5_: 49.7 ± 8.9*^a^ IR: 8.7 ± 2.3*^a^ DR: 9.5 ± 2.8*^a^	*Σ*_1−5_: 38.9 ± 8.8 IR: – DR: 8.6 ± 2.6*	*Σ*_1−5_: 41.6 ± 9.0 IR: – DR: 9.6 ± 2.8**	*Σ*_1−5_: 36.1 ± 7.9 IR: – DR: 7.6 ± 2.0				
F-A-S Test	PRE	21.1 ± 1.0							
	POST	21.7 ± 7.3^a^							
Digit Span	PRE	10.7 ± 2.8							
	POST	10.4 ± 1.9^a^							
Stroop Test	PRE	1st trial: 22 (12–38) 2nd trial: 28.0 ± 6.4							
	POST	1st trial: 20.1 ± 4.8^a^ 2nd trial: 28.1 ± 6.3^a^							
Attentive Matrices Test	PRE		61.9 ± 7.2	62.2 ± 9.4	61.7 ± 4.4				
	POST		64.7 ± 6.8**	64.6 ± 8.3**	64.9 ± 5.1**				
Raven Test	PRE		26.8 ± 4.6	27.7 ± 4.6	25.9 ± 4.7				
	POST		28.0 ± 4.1*	29.4 ± 3.4**	26.5 ± 4.3**				
Drawing Copy	PRE		48.4 ± 11.5	47.4 ± 11.3	49.4 ± 12.1				
	POST		52.8 ± 9.3*	53.1 ± 9.3**	52.6 ± 9.6**				
Verbal Fluency Test	PRE		34.8 ± 11.8	35.7 ± 3.5	33.9 ± 10.3				
	POST		39.3 ± 12.7*	40.7 ± 14.7*	37.9 ± 10.7*				

Data are reported as mean ± SD, or median(range). MoCA, Montreal Cognitive Assessment; S-MMSE, Standardized Mini-Mental Status Examination; ACE-R, Addenbrooke's Cognitive Examination-Revised; T-ICS, Interview for Cognitive Status (administered face-to-face); RAVLT, The Rey Auditory Verbal Learning Test; F-A-S Test, The Oral Fluency Test; CT, Combined training group; AT, Aerobic training group; NCI, no cognitive impaired; CI, cognitive impaired; *Σ*_1−5_, score derived by the sum of RAVLT trials 1 to 5, index of immediate recall ability; IR, immediate recall; DR, delayed recall.

* *p* < 0.05.

** *p* < 0.01.

^a^ Data recalculated from original work results.

## Results

### Search results

**Figure 1:**
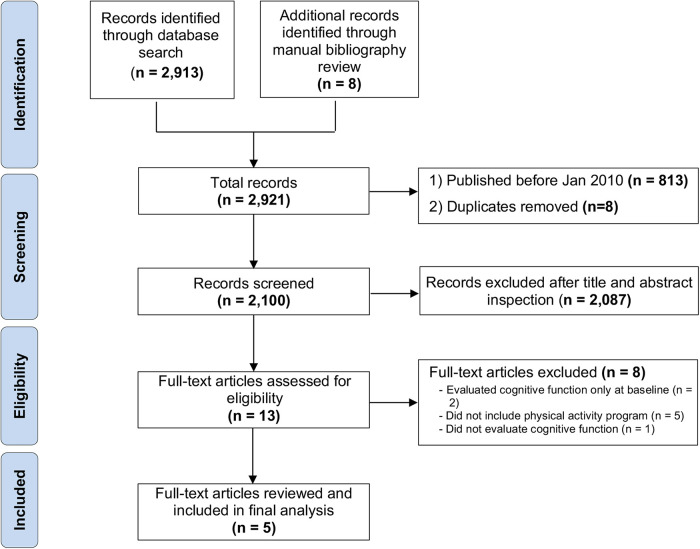
PRISMA flow diagram of identification, screening, and inclusion of eligible articles.

After removing the duplicates and manuscripts published prior to January 2010, the initial search reported a total of 2100 records matching search inclusion criteria. Two-thousand-eighty-seven were removed after completing the title and abstract inspection. A total of 13 full-text articles were screened for eligibility, and five were retained for review analysis. The eight full-text manuscripts were excluded due to missing cognitive function re-evaluation at the end of the intervention (*n* = 2), the physical activity program (*n* = 5), or the cognitive function evaluation (*n* = 1) (Figure 1).

The five manuscripts allocated to final review were a randomized controlled trial (44) and four prospective observational studies (13, 43, 45, 46, 64). Four investigations examined the effects of multidisciplinary PR (13, 43, 45, 46) on cognitive function in COPD population, and the fifth study analyzed the effects of structured exercise training (44). In addition, two of the studies included secondary aims: (1) to determine the prevalence of CI in COPD population (46); and, (2) to assess the time of recovering cognitive function after acute pulmonary exacerbation (45).

### Description of studies cohort characteristics

Two studies included patients with COPD enrolled in outpatient rehabilitation programs (43, 45), one in inpatient PR (13), and another in a nursing home setting (44). One study (46) enrolled two COPD participant groups: (1) patients hospitalized for an acute exacerbation who were followed for 6 weeks after discharge without undergoing PR; and, (2) patients with stable COPD who participated in PR. Considering the inclusion criteria established for this review, only the information relative to this second cohort were allocated to analysis.

Across the five studies, a total of 245 patients with COPD were recruited. Overall, they were older individuals (33% female; 66 ± 8 years old) with moderate to very-severe airflow limitation (GOLD stage 2 to 4). Four studies included male and female participants (13, 43, 45, 46), and one just male individuals (44). In addition, three studies enrolled individuals with a prior diagnosis of CI (13, 45, 46). Three studies provided information about current smoking history: one enrolled only former smokers (44), and the other two reported that 26% (46) and 8% (13) were current smokers. Finally, authors of three of the studies provided data about the presence of comorbidities, and hypertension and metabolic disorders (diabetes ranked first) were the most prevalent (44–46); whereas, one study reported the number of comorbidities (2.6 ± 1.7) without specifying the conditions (13). Another study did not report any specific medical history information about the cohort (43). A detailed summary of the characteristics and design of each study is provided in Table 1. In addition, Table 3 reports the results of the evaluation done at pre and post intervention. To note, while pre intervention data refer to the complete cohort enrolled in each of these studies, the post intervention consider the drop-outs for two studies (45, 46) and therefore results are for those patients who completed the study.

### Exercise-based interventions

Four studies (13, 43, 45, 46) utilized a traditional multidisciplinary PR program, while the fifth study (44) combined either aerobic and/or resistance training with a series of respiratory, balance and mobility exercises (Table 1). The duration of the interventions varied. For the three studies that administered the traditional outpatient PR, the frequency ranged from two (46) to three (43, 45) times a week, for a total of 12 (46) up to a maximum of 36 (43) sessions. For the study administering exercise as part of inpatient PR (13), patients with COPD underwent an 80-minute intervention, four times a week, for 3 weeks, for 12 sessions total. The fifth study (44) evaluated a structured exercise training program outside PR, and participants trained twice a day, for 5 days a week over 4 weeks, for a total of 40 sessions.

Only three studies (13, 44, 45) provided details concerning training workload determination and progression, and details are provided in Table 1. Briefly, for the endurance training, work rate intensity was determined based on the results of a cardiopulmonary exercise test conducted either at 75% of the individual estimated peak work (13) or up to volitional exhaustion (44, 45). For the resistance training, the 1-RM gold standard test was used to set both the upper (44) and lower limb (44, 45) training work rates. One study (13) did not provide specific details about the determination of the work rate for the strength training exercises.

### Cognitive function assessments

Across all studies, a total of 12 neuropsychological tests were utilized to assess cognitive function (i.e., screen for CI) before and after the exercise intervention. A brief explanation of the main purpose of each tool is reported below, and further details are included in Table 2 and Supplementary Figure S1.

Three studies included the Montreal Cognitive Assessment (MoCA (47)), which was administered as a single tool to screen for MCI (45, 46), or in combination with other validated assessments (13). The MoCA screens for MCI assessing multiple domains through completion (8, 47, 48) of 30 tasks. These tasks are scored with a point system, a total score lower than 26 is the cut-off for impairment (see levels of severity in Table 2). The MoCA has high reliability and closely correlates with the Mini-Mental State Examination (MMSE), the most widely used clinical instrument for the detection of dementia (47). The main advantage of using the MoCA is the ability to more accurately screen for MCI in older individuals, given its higher sensitivity compared to MMSE (47). In one study (13), alongside the MoCA, the Standardized Mini-Mental Status Examination (S-MMSE) (50), the Addenbrooke's Cognitive Examination-Revised (ACE-R), and the Interview for Cognitive Status (T-ICS) were administered. The S-MMSE is an updated, briefer version of the MMSE (∼10.5 min on average vs. 13.4 of MMSE; *p* < 0.004), significantly lower variability (86%, *p* < 0.003) and variance compared to the MMSE (50). The examinee completes a series of cognitive function domain-specific tasks; a lower score reflects worse cognitive ability. The ACE-R instrument provides a global and domain-specific evaluation of cognitive function based on 11 tasks; higher scores (total and by domain) reflect higher cognitive functioning (13, 51). The T-ICS is a standardized test originally intended to estimate the severity of CI when formal clinical examinations were not possible (53). The assessment examines several domains of cognition such as orientation (e.g., naming day and time) and memory (e.g., immediate repetition of a set of words read by the examiner). The T-ICS is highly correlated with the MMSE, has a high test-retest reliability, sensitivity, and specificity for the detection of CI in Alzheimer's patients (52), but it is unclear if the same applies for the COPD population.

The other two studies included in our review analysis (43, 44) administered a series of tests aimed to evaluate a single specific cognitive domain (Tables 1 and 2).

Three instruments screened verbal memory skills. The Rey Auditory Verbal Learning Test (Rey-test or RAVLT (65)) is one of the most widely used word verbal learning tests. RAVLT measures verbal learning as well as delayed recall and recognition, among other abilities. This tool is a list-learning paradigm to test recall immediately and 15 min after following auditory presentation of a 15-item word list. The short-term learning and delayed recall represent verbal memory, which progressively decreases with age (65). The Oral Fluency Test (F-A-S) (43), evaluates the fluency of phonemic words (i.e., type of verbal fluency), which taps lexical access capacity and executive control. The F-A-S test requires an individual to orally produce as many words as possible that begin with letters F, A, and S in one minute per letter. The Verbal Fluency test (44), assesses semantic fluency ability (56). For this test, the examinee lists as many words as possible in 2 min, and one point is assigned to each correct word pronounced; a higher score is indicative of better fluency abilities.

Two instruments were used to screen attention-related skills. The Digit Span (66), a subtest of the Wechsler Adult Intelligence Scale and the Wechsler Memory Scale, was administered in one study (43). For the Digit Span scale, the test administrator reads a sequence of numbers to the testee who is instructed to repeat the sequence following the same (forward span) or the reverse (backward span) order to test for attention and working memory, respectively. The Digit Span has a good internal consistency (0.85–0.99) and adequate test-retest reliability (0.75–0.99) in the adult population over 55 years old (67). Similarly, as seen in one study (44), the Attentive Matrices test uses the ability of the testee to retrieve a target number among numeric matrices to assess the level of selective and sustained attention.

The Stroop Color and Word test assesses capacity to inhibit cognitive interference (i.e., ability to maintain focus on a task by removing or not considering information that is irrelevant and otherwise might create disruption in the stream of though (68)). The Stroop test was included in the battery of evaluations administered by Pereira et al. (43). For this assessment, the participant is required to read three different tables as quickly as possible, two representing a “congruous” and one representing an “incongruent” condition (61); a longer time to complete the task reflects poorer performance.

The Raven's test is a multiple-choice exam comprised of a series of visual pattern matching and analogy problems without representational designs, to assess the abstract reasoning skills (62). Briefly, the participant is presented with 12 incomplete figures and one point is assigned for correct identification of each missing piece. A higher score on the 36-point scale reflects better performance.

The Drawing Copy Test evaluates praxis skills, i.e., the ability to execute movements. In this test the participant is required to complete two drawing tasks (13, 63); a greater score indicates a better praxis ability.

### Effects of exercise interventions on cognitive function in COPD

Overall, all five studies reported improvements in various domains of cognitive function following the exercise interventions. Table 3 summarizes the results at baseline (pre) and post-intervention.

Improvements in global cognition were reported in three studies administering the MoCA test following exercise trainings embedded in PR (13, 45, 46). Bonnevie et al. (45) found that the mean MoCA score increased by 3 points after 8 weeks of intervention (Pre: 22 (interquartile range or IQR 20 to 26); post 25 (IQR 23 to 28); *p* < 0.01) (16). Participants who were diagnosed with MCI at baseline showed a statistically significant improvement at the end of PR (from 21 (IQR 20 to 24) to 22 (IQR 20 to 26); *p* < 0.01), which was sustained at 3 months of follow-up (24 (IQR 21 to 26), *p* < 0.01) (45). A statistically significant increase (Δscore = 1.60 points; *p* = 0.004) was also reported for the 42 patients with COPD who completed the 6-week intervention conducted by France et al. (46). Specifically, the reported increase was found in 25 participants presenting with baseline CI. Similar to Bonnevie et al. (45) no significant changes were observed among the participants with NCI at the time of study enrollment (46). On the contrary, minor improvements (Δscore = 0.50 points) were reported for the NCI group in Andrianopoulos et al. (13) and no changes were calculated for the MoCA score of the 25 COPD who had CI at the inpatient-PR program at intake (Δscore = 0.20 points). Nevertheless, larger improvements were seen in the CI group compared to the NCI group based on the additional three screening tools administered by Andrianopoulos et al. (13) assessing global cognition. Despite modest score changes in the S-MMSE (CI: Δscore = 0.80 points; NCI: Δscore = 0.50 points), ACE-R (CI: Δscore = 4.70 points, NCI: Δscore = 1.60 points) and T-ICS (CI: Δscore = 1.30 points; NCI: Δscore = 1.00 points) scores, both the NCI and CI groups reported a statistically significant improvement at post-intervention (*p* < 0.05).

Contrasting results for immediate recall ability were found in the RAVLT in two studies (43, 44). The sum of the five trials score (i.e., *Σ*_1–5_ results in Table 3), which refers to immediate recall ability, increased by 6.23 points (*p* < 0.05) in patients with COPD who completed 3 months of PR, compared to no to minimal changes in the group of patients who underwent either a combination of resistance and aerobic training (Δscore = 0.29 points) or an aerobic-centered exercise program (no change) (44). Pereira et al. (43) found that the single trial for short or immediate recall ability (IR) resulted in an improvement in participant performance at the end of the intervention (Δscore = 1.14 points). Consistent positive improvements were found in the delayed recall (DR) as measured by the RAVLT at post intervention (43, 44). In Aquino et al. (44), the increase in delayed recall ability was almost twofold in the COPD group assigned to the combined training (Δscore = 1.35 points) compared to patients who completed the aerobic-centered program (Δscore = 0.64 points).

Additionally, Pereira et al. (43) assessed the capacity to inhibit cognitive interference with the Stroop test. At post intervention, the 34 moderate to very-severe COPD participants decreased on average 2 s the time to complete the reading/naming tasks during the first of two test trials, but registered no changes in response time to complete the second trial (0.12 s longer response time). Similarly, a positive, modest change was found for the phonemic fluency on the F-A-S test. Although no difference in the number of words produced was obtained, the large standard deviation at the end of the intervention indicates that a subset of the patients improved their performance in the organization of verbal processing. Lastly, non-significant changes were found for the attention, concentration and memory control on the Digit span test (43). This cognitive domain was also evaluated by Aquino et al. (44) with the Attentive and Matrices test. The latter study compared two different training paradigms, i.e., aerobic vs. the combination of aerobic and resistance exercises (44), randomizing 14 former smokers with COPD to each group. Independent of the training modality, significant improvements in selective and sustained attention were reported in the 28 participants (Δscore = 2.78 points; *p* < 0.01). However, aerobic-centered training induced a greater improvement (Δscore = 3.15 points; *p* < 0.01) than the combined modality (Δscore = 2.43 points; *p* < 0.01). Similar improvements were found for abstract reasoning ability (Raven test Δscore = 1.17 points; *p* < 0.05), praxis ability (Drawing Copy test Δscore = 4.46 points; *p* < 0.05), and semantic fluency ability (Verbal Fluency test Δscore = 4.50 points; *p* < 0.05), for the entire cohort. Greater improvements (*p* < 0.05) derived from the combined resistance + aerobic exercises (CT) training method compared to the aerobic exercise program (AT) in these three cognitive function abilities (i.e., abstract reasoning, praxis abilities, and semantic fluency).

## Discussion

The present work undertook a scoping review of the effects of exercise interventions on cognitive function performance in older individuals with stable COPD, aiming to provide comprehensive information on (1) the types of exercise-based training interventions and cognitive function tests administered to patients with COPD, and (2) the magnitude of changes in cognitive processes and abilities in stable COPD that underwent an exercise-based intervention. Thirteen full-text manuscripts were assessed for eligibility, and five studies met the inclusion criteria. Despite the high prevalence of this condition reported in COPD (10%–61%; (5–9)) and the description of specific physiological changes leading to CI in the COPD population, the limited number of sources allocated to final review confirms that routine evaluations of CI are scarce in this population. The presence of systemic inflammation secondary to hypoxic stress, in particular, has been proposed as a major contributing factor for neuronal injury (e.g., stroke, cerebral edema) that results in neuropsychological deficits in COPD (14, 69). Given that neuropsychological impairments may adversely impact patient disease management such as symptom monitoring, medication adherence (19) and acquisition and memory of novel medical information, it is surprising that cognitive functioning is not routinely assessed in patients with COPD. Although medical assessments (e.g., functional and diffusion tension imaging magnetic resonance) may not be a preferred choice due to the high costs and specific skills required to administer and evaluate the test, the neuropsychological screening tools discussed in this review (Table 2 and Supplementary Figure S1) are brief (4 to 12 min) to complete and score, and require easy obtainable training to ensure proper administration and interpretation of findings. However, our analysis revealed the heterogeneity of cognitive screenings with varying psychometric properties that have been administered to the COPD population. These factors limit the conclusions around the effects of exercise on patients diagnosed with COPD, and the determination of the most effective strategies to improve cognitive function abilities. This observation echoes the perspectives of Desveaux et al. (36) and Blackstock et al. (35), and reiterate the urgency of uniform cognitive function screening tools to evaluate patients with COPD.

Nevertheless, our review suggests that exercise training, administered as a component of the traditional PR program or as an independent structured intervention, may improve cognitive abilities in older people with stable COPD, particularly in those patients presenting with signs of CI prior to the interventions. These latter findings are similar to those in Ohman et al. systematic review (70), which described the positive effects of physical activity on executive function, attention, and delayed recall abilities in older individuals with MCI.

We discuss below the main improvements in five of the six basic essential neuropsychological domains of cognition (9) highlighting the positive changes we determined with our analysis. Furthermore, given that three of the studies (13, 45, 46) included assessments that yielded a single score that is a composite of multiple cognitive domains (i.e., MoCA, S-MMSE, ACE-R, T-ICS), these results are discussed in a separate paragraph at the end (*Global cognition*).

### Language

Language ability is generally determined by assessing the semantic and phonemic fluency sub-categories with tasks involving the verbal naming of as many words from a single category as possible in a defined length of time, e.g., 60 s (71). These types of tests are often included within a larger battery of cognitive assessments with the aim of detecting signs of cognitive decline (71). To evaluate language ability, a primary objective is to determine the strategies the individual uses to create and select the appropriate responses, which also depend on distinct memory processes and are therefore tested evenly (72). Both semantic and phonemic fluency depend on partially shared (e.g., energization, self-monitoring, attention, processing speed, language) and partially distinct (e.g., search strategy, semantic vs. phonological memory) cognitive processes (72). These cognitive skills were assessed by two of the five studies included in our analysis. Aquino et al. (44) directed participants to list familiar words across four categories (colors, animals, fruits, names of cities) within a time frame of 2 min. After 4 weeks of a structured exercise intervention, significant improvements (*p* < 0.05) were observed in fluency, with a larger increase seen in patients randomized to the CT training compared to AT. Using a similar testing procedure (i.e., F-A-S test), Pereira et al. (43) reported a smaller improvement in a group of 34 COPD undergoing the exercise intervention as part of the PR. These findings suggest that the PR intervention might be less effective in improving verbal fluency than the structured exercise training proposed by Aquino et al. Since cognitive dysfunction is associated with the severity of the airflow limitation (14, 35), the poorer outcome reported by Pereira et al. (43) could be partially explained by the sample which was largely comprised of patients with severe and very-severe COPD (*n* = 23) compared to Aquino et al. (44), study that enrolled patients with mild-to-moderate airflow limitation. However, since Pereira et al. (43) did not report F-A-S test scores as function of the severity stages of the COPD disease, our hypothesis should be verified by future studies stratifying test scores as a function of the four COPD GOLD stages, to provide clarity on the association between language abilities and disease severity.

### Memory

Memory constitutes one of the six main domains of cognitive function, and it is subdivided into eight specific abilities including delayed memory, encoding memory, long- and short-term memory, prospective memory, verbal memory, and reasoning memory (9). Both studies that primarily assessed memory performance with the RAVLT (43, 44), found changes in both short-term and delayed-recall memory following PR or a structured exercise intervention. In the study by Aquino et al. (44), neither the CT nor the AT program improved immediate recall. In contrast, the CT condition induced a significant increase (*p* < 0.01) in delayed recall, an improvement that was almost twice that of the AT group (44). Interestingly, based on in Pereira et al.'s study (43), we calculated a larger increase in the immediate recall (Δ*Σ*_1–5_ score = 6.23 points; *p* < 0.05) than the delayed recall ability (Δ*Σ*_1–5_ score = 1.14 points, *p* < 0.05). Furthermore, younger age and male sex were factors that affected the performance in the delayed recall task in Pereira et al. cohort (*p* = 0.004 and 0.009, respectively). Contrary to the abovementioned *Language* findings, in the context of *Memory* both types of interventions (i.e., structure exercise intervention and PR) improved the ability to recall words short-term (i.e., after 15 min), but the PR intervention seems to be more effective in improving immediate memory recall performance. Overall, further research is warranted to examine the effects of exercise on immediate and short-term memory as the current literature is scarce, and there is no description of cognitive function across the GOLD severity stages.

### Attention

Attention or attention/concentration relates to specific functions such as alternating attention, selective attention, sustained attention, divided attention, and processing speed (9). This dimension was evaluated by the Stroop test, which mainly measures focused attention, and the Digit Span test, which evaluates attention and concentration, mental control and short-term memory (43); and through the Attentive Matrices Test, specific for selective and sustained attention evaluation (44).

The 12-week intervention designed by Pereira's group resulted in improvement in the performance on both tests. Specifically a increase was obtained in the task completion time for the Stroop (∼2 s decrease in task completion time), which was independent of sex (*p* = 0.118) (43). Similarly, Aquino et al. (44) reported significant improvements at post intervention (*p* < 0.01), with greater gains induced by the AT compared to the CT training modality. Overall, these findings reinforce the notion that exercise, regardless of the modality of delivery (PR or structured exercise training) or content (aerobic, resistance, or combined), positively affects the cognitive domain of attention.

### Executive functions

*Executive functions* is an umbrella term to refer to a cognitive function domain that includes ten subcategories such as reasoning, flexible problem solving, planning, decision making, and mental flexibility (9). Abstract reasoning (non-verbal logical reasoning) and problem-solving ability were evaluated by the Raven's progressive matrices test in one study (44). Similar to other screening tools that evaluate inductive reasoning and diagrammatic reasoning, the Raven is a non-verbal screening instrument requiring the examinee to recompose a series of figures with a missing component. Overall, at post intervention, exercise training improved executive function abilities, with a superior outcome obtained by the patients with COPD who underwent the CT (Δscore = 1.7 points, *p* < 0.01) compared to those that were assigned to the aerobic-centered program (Δscore = 0.6 points, *p* < 0.01). These findings indicate that adding resistance exercises to the more traditional training paradigm that focuses on aerobic exercises improves executive function abilities in patients with stable COPD.

### Praxis

Praxis abilities are the capacity of an individual to perform skilled or learned movements (73). Most of the assessments available to assess praxis are based on drawing or copying of graphical elements. In general, drawing is a complex ability that integrates several cortical and subcortical areas (74), and hence, test scores that fall below normal limits are suggestive of cerebral damage and cognitive dysfunction (75). Aquino et al. (44) conducted the only study to measure praxis using a specific instrument, i.e., the Drawing Copy test. Participants were instructed to copy a drawing, and the score was based on the adherence to the original drawing. Interestingly, similar to recall ability and verbal fluency mentioned above, the CT modality produced greater improvements in praxis compared to the AT program, despite both statistically significant compared to pre-intervention (*p* < 0.01). Aquino and colleagues (44) posit that improved cognitive performance may result from a more cognitively complex task of free-weight resistance exercises vs. cyclic exercises, which are the core components of aerobic focused training. The impact of strength training on cognition was evaluated in a recent meta-analysis (76), which suggested that weight training may lead to improved sustained attention as a function of continual focus on the repetitive task of weight lifting in adults age 50 and older. Further evidence is needed to confirm the effects of regular strength training on praxis abilities in COPD.

### Global cognition

As previously mentioned, four instruments (MoCA, S-MMSE, ACE-R, T-ICS) were administered to assess presence and severity of CI by three studies included in our analysis (13, 45, 46). While these tools include a series of tasks each one tapping to specific cognitive abilities, the analysis of the test results returns a composite or total score reflecting global cognition performance.

Independently of CI, at post-intervention, the MoCA score of the participants in Bonnevie et al.'s study (45) increased twofold compared to those enrolled in France et al.'s investigation (46) and six-times compared to both patient groups enrolled in Andrianopoulos et al.'s study (13). The improvements were consistently larger for the patients with CI than NCI in all three studies. Overall, at post intervention, patients with COPD reached values close to the lower normal range of MoCA score (i.e., 26) confirming that regular exercise activity is crucial to maintaining or improving cognitive abilities in COPD. In addition, when patients from Bonnevie et al.'s study (45) underwent a third evaluation at 3 months following PR, the improvement in global cognition increased in those with a pre-existing diagnosis of CI. The authors suggested that the overtime positive benefits observed in these participants might have been residual effects of the intervention, but this assumption awaits confirmation by further longitudinal studies.

In addition to the MoCA, Andrianopoulos et al. (13) administered the S-MMSE, ACE-R, and the T-ICS. The improvements induced by the inpatient PR program were modest but statistically significant (*p* < 0.05; Table 3) in the three tests. Interestingly, upon merging the results of these tests with the MoCA and the combined scores expressed in function of the six main cognitive domains, the patients with CI showed a significant improvement in fluency and visuospatial abilities (*p* < 0.05), while the NCI group primarily increased performance in language and executive skills (*p* < 0.05) (13).

### Limitations

This scoping review has some limitations. A study protocol was not published in advance; therefore, the methods were not peer-reviewed prior to conducting the search. We selected only manuscript published in English, excluding gray literature, and the search strategy we applied, despite using multiple terms to describe the main concepts (Supplementary Table S1), might potentially have missed relevant published information. Nevertheless, the body of work published in the past decade relative to the evaluation and management of cognitive function in patients with COPD is indeed quite limited. We also did not perform a formal methodological quality assessment of the included manuscripts.

Furthermore, two of the five studies allocated to review (45, 46) assessed cognitive function with a single instrument, i.e., the MoCA, while the other three (43, 44) included multiple instruments to evaluate specific cognitive function domains (Supplementary Figure S1). Moreover, improvements in cognitive functions may be influenced by duration, intensity, frequency, total number of exercise sessions and the exercises modality (e.g., aerobic, resistance, combination). The different study designs and methods across studies limit the conclusions that can be drawn from our analysis, possibly misleading the interpretation of the results related to the specific neuropsychological domains. Further studies should determine the optimal training paradigm for reaching the maximum cognitive function gain, and seek to establish the appropriate “exercise dose” for each specific cognitive function domain.

Relatedly, while the overall direction of change is positive, the clinical significance of these findings is difficult to determine. Interpreting the changes on neuropsychological tests is related to the psychometric properties of a given test for a given population. Multiple approaches have been suggested to evaluate the clinical significance of change scores, such as the regression-based change method (77), minimal clinically important difference (MCID) (78), and standard error of difference (67). Reliable score changes for Digit Span and other neuropsychological tests are available for individuals with Alzheimer's disease, schizophrenia and chronic alcohol abuse (67) or the healthy adult population (77), but not yet for patients with COPD. This problem could potentially be addressed if the raw data from the studies included in our review were available to conduct further analysis, but that would also imply that each test was repeated at least twice to test for reliability and internal consistency (67). Therefore, establishing if a training intervention or the progression of the COPD disease has a MCID in cognitive function performance is not currently possible and deserves additional research.

In addition, only one study (43) enrolled a group of age-matched, healthy subjects. Between group comparison with peer healthy subjects would further define the effects of the intervention in COPD. Of note, despite the intrinsic advantage of these tests in requiring short time for administration and scoring, none of the studies included in our review administered the cognitive function tests on *ad interim* basis, such as when the training work rate was adjusted based on participants progression. As a result, future research should investigate the acute or chronic effects of exercise on ameliorating various domains of cognitive function in COPD over time. Finally, in 2019 Lavoie et al. (79) published an elegant study evaluating the effects of self-management behavioral modification with and without bronchodilators and/or exercise training, reporting cognitive performance improved with increased physical activity and exercise capacity. The aim of the present review was to isolate the effects of a structured exercise intervention on cognitive performance in patients with stable COPD, and the study by Lavoie et al. (79) did not meet criteria for inclusion in our analysis. Lavoie's investigation is highly relevant and adds a significant contribution to the topic, and future studies comparing the effects of behavioral, pharmacological and exercise interventions are warranted to further clarify the independent effects of each intervention on cognitive performance in patients with COPD.

Despite the limitations, cognitive dysfunction is prevalent in the COPD population and this scoping review shed light on the important role exercise-based interventions play in maintaining and potentially improving cognitive function. Most of the studies we considered delivered exercise within a traditional PR program. Pulmonary rehabilitation is a learning environment that provides education and practical strategies for patients with lung disease to implement in their daily lives (35). However, due to the multidisciplinary nature of PR programs, it is difficult to quantify the benefits derived from exercise from the education and/or other program components. Therefore, there is a clear need to further evaluate the effects of PR on cognitive function by comparing traditional PR with programs that adopt screening tools, exercise and education aiming for cognition ability training in patients with COPD.

### Conclusions

In summary, exercise interventions, included in the traditional PR program or as a specific training regimen, improve test scores in several areas of cognitive functioning in older patients with stable COPD. Larger benefits seem achievable with a combination of resistance and aerobic exercises. However, despite the wide prevalence of CI among patients with COPD, no guidelines are available on which and how properly administer cognitive function evaluations, or the strategies to adopt to improve cognitive function (35). It is important for future studies to focus on validating the optimal battery of tests to evaluate cognitive function comprehensively in this population. Furthermore, since many cognitive assessments are brief (4 to 12 min) to complete and score, properly trained field experts, like pulmonary therapists, should include these evaluations on a routine basis to screen for CI.
