# Supplementary material for: The role of structured exercise interventions on cognitive function in older individuals with stable Chronic Obstructive Pulmonary Disease: A scoping review
Source: Front Rehabil Sci. 2022 Oct 31;3:987356. doi: 10.3389/fresc.2022.987356 (PMC9659625; doi:10.3389/fresc.2022.987356)
Supplement: Supplementary file 1 [file Table1.pdf]

SUPPLEMENTARY MATERIAL

Table S.1: Concepts and terms used for identification of studies during electronic search

Concept 1	Concept 2	Concept 3	Concept 4
Cognitive function	Chronic obstructive pulmonary disease	Physical activity	Smoker
Cognitive impairment	COPD	Exercise	Tobacco users
Cognitive decline	Emphysema	Activity	Smoking
Cognitive dysfunction	Chronic bronchitis	Aerobic exercise	
Cognitive defect	Obstructive airways disease	Aerobic training	
Mental health	Chronic lung diseases	Resistance exercise	
		Resistance training	
		Pulmonary rehab	
