# Supplementary material for: The role of structured exercise interventions on cognitive function in older individuals with stable Chronic Obstructive Pulmonary Disease: A scoping review
Source: Front Rehabil Sci. 2022 Oct 31;3:987356. doi: 10.3389/fresc.2022.987356 (PMC9659625; doi:10.3389/fresc.2022.987356)
Supplement: Supplementary file 2 [file Suppl_S2.pdf]

SUPPLEMENTARY MATERIAL

Supplementary S2.: Search Strategy

- The database searches are a combination of keyword and controlled vocabulary terms to provide the most comprehensive coverage.
- The Ovid MEDLINE codes are captured in the search and a combination of keyword and MeSH terms have been used to provide comprehensive coverage.

Example search strategy for Medline (Ovid)

#	Search	Records retrieved
1	(cognitive function or cognitive impairment or cognitive decline or cognitive dysfunction or cognitive defect or mental health).mp.	384304
2	(chronic obstructive pulmonary disease or COPD or emphysema or chronic bronchitis or obstructive airways disease or chronic lung disease).mp.	121303
3	(physical activity or exercise or activity or aerobic exercise or aerobic training or resistance exercise or resistance training or pulmonary rehab$).mp.	3421830
4	(smoker or tobacco user$ or smoking or smok$).mp.	367752
5	1 and 2	1133
6	1 and 2 and 3	247
7	1 and 4	8144
8	1 and 3 and 4	1796
9	1 and 3 and (2 or 4)	1993
10	limit 6 to (English language and yr="2010 - 2021")	182
11	limit 8 to (English language and yr="2010 - 2021")	1289
12	limit 9 to (English language and yr="2010 - 2021")	1431
13	((cognitive function or cognitive impairment or cognitive decline or cognitive dysfunction or cognitive defect or mental health) and (physical activity or exercise or activity or aerobic exercise or aerobic training or resistance exercise or resistance training or pulmonary rehab$) and (chronic obstructive pulmonary disease or COPD or	1635

	emphysema or chronic bronchitis or obstructive airways disease or chronic lung disease or (smoker or tobacco user$ or smoking or smok$))).ti,ab.	
14	limit 13 to (English language and yr="2010 - 2021")	1174
15	((cognitive function or cognitive impairment or cognitive decline or cognitive dysfunction or cognitive defect or mental health) and (chronic obstructive pulmonary disease or COPD or emphysema or chronic bronchitis or obstructive airways disease or chronic lung disease) and (physical activity or exercise or activity or aerobic exercise or aerobic training or resistance exercise or resistance training or pulmonary rehab$)).ti,ab.	216
16	limit 15 to (English language and yr="2010 - 2021")	158
17	((cognitive function or cognitive impairment or cognitive decline or cognitive dysfunction or cognitive defect or mental health) and (physical activity or exercise or activity or aerobic exercise or aerobic training or resistance exercise or resistance training or pulmonary rehab$) and (smoker or tobacco user$ or smoking or smok$)).ti,ab.	1459
18	limit 17 to (English language and yr="2010 - 2021")	1047
19	2 and 4	21201
20	2 and 3 and 4	2934
21	limit 19 to (English language and yr="2010 - 2021")	11655
22	limit 20 to (English language and yr="2010 - 2021")	1710
