# Supplementary material for: The role of structured exercise interventions on cognitive function in older individuals with stable Chronic Obstructive Pulmonary Disease: A scoping review
Source: Front Rehabil Sci. 2022 Oct 31;3:987356. doi: 10.3389/fresc.2022.987356 (PMC9659625; doi:10.3389/fresc.2022.987356)
Supplement: Supplementary file 3 [file Suppl_Figure_S1.pdf]

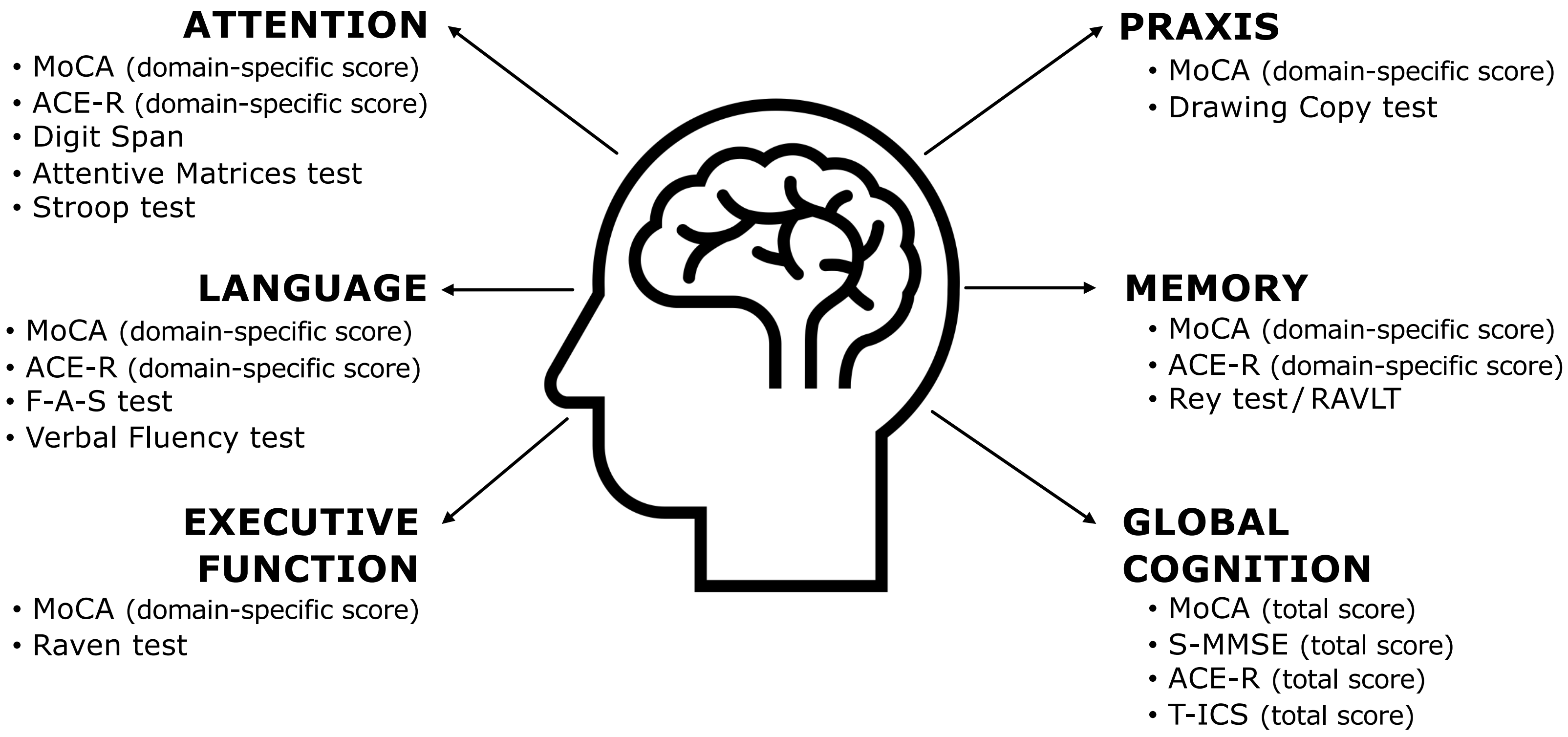

Supplementary Figure S1. Graphical representation of the six cognitive function domains assessed by the five studies included in the scoping review. The instruments used to assess each domain are listed.
